# Characterization, Antiplasmodial and Cytotoxic Activities of Green Synthesized Iron Oxide Nanoparticles Using *Nephrolepis exaltata* Aqueous Extract

**DOI:** 10.3390/molecules27154931

**Published:** 2022-08-03

**Authors:** Faisal Nadeem, Fozia Fozia, Madeeha Aslam, Ijaz Ahmad, Shakeel Ahmad, Riaz Ullah, Mikhlid H. Almutairi, Lotfi Aleya, Mohamed M. Abdel-Daim

**Affiliations:** 1 Department of Chemistry, Kohat University of Science & Technology, Kohat 26000, Khyber Pakhtunkhwa, Pakistan; organicchm192@gmail.com (F.N.); madeehaaslam06@gmail.com (M.A.); saislamian@yahoo.com (S.A.); 2 Biochemistry Department, Khyber Medical University, Institute of Medical Sciences, Kohat 26000, Khyber Pakhtunkhwa, Pakistan; drfoziazeb@yahoo.com; 3 Department of Pharmacognosy, College of Pharmacy, King Saud University, Riyadh 11451, Saudi Arabia; 4 Department of Zoology, College of Science, King Saud University, P.O. Box 2455, Riyadh 11451, Saudi Arabia; malmutari@ksu.edu.sa; 5 Chrono-Environnement Laboratory, UMR CNRS 6249, Bourgogne, Franche-Comté University, CEDEX, F-25030 Besançon, France; lotfi.aleya@univ-fcomte.fr; 6 Pharmacology Department, Faculty of Veterinary Medicine, Suez Canal University, Ismailia 41522, Egypt; abdeldaim.m@vet.suez.edu.eg

**Keywords:** green synthesis, *Nephrolepis exaltata*, FeO NPs, characterization, antiplasmodial and cytotoxic activities

## Abstract

The use of non-toxic synthesis of iron oxide nanoparticles (FeO NPs) by an aqueous plant extract has proven to be a viable and environmentally friendly method. Therefore, the present investigation is based on the FeO NPs synthesis by means of FeCl_3_·6H_2_O as a precursor, and the plant extract of *Nephrolepis exaltata* (*N. exaltata*) serves as a capping and reducing agent. Various techniques were used to examine the synthesized FeO NPs, such as UV-Visible Spectroscopy (UV-Vis), Fourier Transform Infrared Spectroscopy (FT-IR), X-ray Diffraction (XRD), Scanning Electron Microscopy (SEM), and Energy Dispersive X-ray (EDX). The FT-IR studies were used to identify different photoactive biomolecules at 3285, 2928, 1415, 1170, and 600 cm^−1^ in the wavenumber range from 4000 to 400 cm^−1^, indicating the -OH, C-H, C-O, C-C, and M-O groups, respectively. The XRD examination exhibited crystallinity, and the average diameter of the particle was 16 nm. The spherical nature of synthesized FeO NPs was recognized by SEM images, while the elemental composition of nanoparticles was identified by an EDX spectrophotometer. The antiplasmodial activity of synthesized FeO NPs was investigated against Plasmodium parasites. The antiplasmodial property of FeO NPs was evaluated by means of parasite inhibitory concentration, which showed higher efficiency (62 ± 1.3 at 25 μg/mL) against Plasmodium parasite if compared to plant extracts and precursor. The cytotoxicity of FeO NPs was also assessed in human peripheral blood mononuclear cells (PBMCs) under in vitro conditions. The lack of toxic effects through FeO NPs keeps them more effective for use in pharmaceutical and medical applications.

## 1. Introduction

Green synthesized metallic nanoparticles are reported by many researchers, which showed enhanced applications. These applications of nanoparticles are referred to their large surface area and a high fraction of surface atoms [1,2,3,4,5,6].

Magnetic nanoparticles are a specific form of nanoparticle that is affected by magnetic field gradients and are frequently used as the core of nano-biomaterials. Due to their capabilities and low side effects, different kinds of metal oxide and metallic nanoparticles such as silver, iron, nickel, gold, copper oxide, and zinc oxide are very attractive to a wide range of morphological features [7,8]. Iron oxide nanoparticles (FeO NPs) have attracted a lot of attention among numerous metal and metal oxide nanoparticles because of their exclusive properties such as magnetic properties, easy separation methodology, and high surface area [9]. The FeO NPs have numerous applications in the field of biomedicine [10], diagnostic [11], bioremediation [12], and drug delivery [13]. Various polymorph structures of FeO NPs have been investigated intensively due to their widespread applications throughout technological innovation and contemporary science. The most commonly used FeO NPs are magnetite (Fe_3_O_4_, ferrimagnetic, superparamagnetic with size less than 15 nm), hematite (α-Fe_2_O_3_, weakly ferromagnetic or antiferromagnetic), maghemite (γ-Fe_2_O_3_, ferrimagnetic), and FeO (wustite, antiferromagnetic), of which magnetite and maghemite exhibit an outstanding physicochemical property since its biocompatibility has already been proven [14]. Several chemical and physical approaches can be used to synthesize FeO NPs, such as thermal decomposition [15], co-precipitation [16], and chemical vapor deposition [17]. However, the majority of these methods have several drawbacks, including high costs, the need for specialized equipment, high energy consumption, and low productivity. These processes also have negative environmental and health consequences due to the use of flammable, toxic, and corrosive chemicals, as well as the use of organic solvents as dispersing, stabilizing, reducing, and capping agents [18] in order to remove the assumptions of traditional methods and develop the nanoparticle production through more sustainable, efficient, and environmentally friendly processes. Therefore, biological sources have emerged as a viable alternative to traditional methods. According to recent literature, different plant extracts, such as *Agrewia optiva* and *Prunus persica* [19], *Salvia officinalis* [20], and *Mimosa pudica* [21], were used for FeO NPs, which act as a capping and reducing agent.

Malaria is a parasitic disease spread by the bite of infected female Anopheles mosquitoes. Malaria is a parasitic disorder that is among the top causes of mortality worldwide. Despite the best efforts of the World Health Organization (WHO) to eradicate it, the disease is still one of the major health issues for people living in developing countries. The highest prevalence of this disorder is concerning, and exacerbating the problem is the fact that parasitic infection does not trigger a sterilizing immune reaction, ultimately resulting in vaccination failure [20]. The treatment option for this parasitic infection is chemotherapy, where antiplasmodial drugs are available as a discrete and aggregated form of treatment that remains the primary therapeutic approach to control the disease of malaria. Different drugs are used to treat parasitic diseases; it may be either in a single category or their respective classes which include Amodiaquine (AQ), Chloroquine, (CQ 4-aminoquinolines), Quinine (Cinchona alkaloid), and Piperaquine. However, non-artemisinin antiplasmodial drugs have some drawbacks, such as reduced efficacy, high cost, and the potential for toxicity, which leads to low patient compliance. However, the most serious threat to antiplasmodial drugs is the existence of resistance [21]. Mostly, herbal medicine has remained essentially the keystone of malaria treatment for thousands of years. The *Cinchona* tree that belongs to Rubiaceae family, where the antiplasmodial drug Quinine was discovered first from their barks, acts as traditional medicine. Early in the sixteenth century, a *Cinchona* bark extract was used to diagnose different malaria diseases [22]. Therefore, medicinal plants have been recommended to control Plasmodium parasites and resist the antiplasmodial drugs and also exhibit a significant contribution to the treatment of malaria disease.

Thus, the current investigation was based on a green source synthesis of FeO NPs using plant extract of *N. exaltata* that was not reported earlier. The plant *N. exaltata* is commonly known as sword fern or Boston fern, which belongs to the Nephroleideaceae family. It is found in tropical regions of the West Indies, Mexico, South America, Central America, Africa, Polynesia, and Pakistan. The lush green terrestrial plant has a non-flowering and evergreen plant that produces spores on the bottom edge of its dark brown to black color leaves. *N. exaltata* is an ornamental plant that thrives in moist, shady environments and has the ability to remove pollutants from air, soil, or water. It also absorbs gases through its leaves and roots; therefore, the leaf mask of plant *N. exaltata* has been reported to protect of the respiratory tract against volatile organic compounds [23]. *N. exaltata* plant is known to be non-toxic and has been used to treat fever, cough, and antibacterial, antifungal, and cytotoxic activities [24]. The phytochemical analysis revealed that a large number of biomolecules such as saponins, steroids, tannins, alkaloids, and phenols are present in plant extract, which mainly serves as a reducing and capping agent for FeO NPs synthesis [25]. Therefore, this work was carried out to synthesize FeO NPs using *N. exaltata* aqueous plant extract and then to explore the antiplasmodial and cytotoxic activities of the synthesized FeO NPs. The synthesis of FeO NPs using an aqueous extract of this plant has not been reported in the literature, and in this article, we are reporting it for the first time. Furthermore, different strategies such as EDX, SEM, XRD, FT-IR, and UV-Vis Spectrophotometry were employed to examine the elemental composition, geometry, morphology, and optical properties of synthesized FeO NPs. In addition to that, the present research was designed to evaluate the potential applications in the biomedical field through green synthesized FeO NPs for antiplasmodial activity and also evaluate its cytotoxicity by using an MTT assay to assess its biocompatibility. 

## 2. Materials and Methods

### 2.1. Plant Collection and Extraction 

*N. exaltata* fresh plant was collected from the hilly areas of District Kohat, Khyber-Pakhtunkhwa, Pakistan. To eliminate the dust particles and other contaminants, the plant was surface cleaned with distilled water and shade-dried at room temperature, and 20 g of the finely cut plant was heated for 30 min in a beaker containing 200 mL of distilled water. The aqueous extract of the plant was allowed to cool, then filtered using Whatman filter paper No. 1 before being stored in the refrigerator.

### 2.2. Green Synthesis of FeO NPs 

The green synthesis procedure was used to synthesize FeO NPs. The synthesis of FeO NPs was achieved by mixing FeCl_3_·6H_2_O and *N. exaltata* plant extract in volume ratios of 10 mL of 0.01 M FeCl_3_·6H_2_O aqueous solution to 90 mL *N. exaltata* plant extract (1:9 *v*/*v*). The pH of the reaction mixture was adjusted to pH 8 and heated at 60 °C, then stirred continuously for 2 h at 200 rpm using a rotatory orbital shaker. The formation of nanoparticles was confirmed by the color of the solution, which changed from pale yellow to dark brown within a few minutes. After that, the reaction mixture was examined by a UV-Vis spectrophotometer. The best UV-Vis peak was obtained after 30 min of the reaction. The synthesized FeO NPs were centrifuged at 6000 rpm for 20 min and rinsed three to four times with distilled water and ethanol to remove unbounded molecule. The precipitate was dehydrated under vacuum at 40 °C temperature to obtain powder form, which was then used for further analysis [26].

### 2.3. Characterization of FeO NPs

Different strategies including UV-Vis, FT-IR, XRD, SEM, and EDX analysis were used to characterize the synthesized FeO NPs.

#### 2.3.1. UV-Vis Spectroscopy 

The reaction mixture was analyzed at the wavelength ranges of 200–800 nm by using a UV-Vis Spectrophotometer. The synthesis of FeO NPs arises through an iron ion reduction process and then followed at regular intervals by monitoring the UV-Vis spectra of a solution until no changes in absorbance are visible.

#### 2.3.2. FT-IR Analysis 

The FT-IR analysis is used to study bond details of compounds. The potassium bromide pellet method was used to determine the bonding type and strength of both plant extract and FeO NPs within the range of 4000–400 cm^−1^ at 4 cm^−1^ resolutions. In this experiment, 350 mg of fine KBr (potassium bromide) was immediately mixed with 1–3 mg of FeO NPs, which is relocated to a disc containing a barrel diameter of 13 mm and pressed for 1–2 min at 12,000 psi. The FT-IR spectrophotometer was employed to investigate the biomolecules present in plant extract at 1 mm thick re-crystallized KBr disc.

#### 2.3.3. XRD Analysis

The crystallinity of metallic nanoparticles was studied by employing an X-ray diffractometer (30 kV/30 mA) along with a Ni filter and CuKa radiation source. Under experimental conditions, the data of all powder forms nanoparticles in a sample of X-ray diffraction were obtained at 2θ angle and ranging from 10 to 80°.

#### 2.3.4. SEM Analysis

The shape, size, and morphological properties of nanoparticles were examined by using the SEM technique. A fine powder of FeO NPs was used to generate the image, which was taken with a JEOL JSM-6510LV scanning electron microscope at a 20 kV accelerating voltage.

#### 2.3.5. Energy Dispersive X-ray (EDX)

The EDX technique was carried out to detect the shape, surface morphology, and elemental compositions of the synthesized FeO NPs at an accelerating voltage of 20 keV.

### 2.4. Antiplasmodial Activity

#### 2.4.1. Sample Collection

The malaria-positive samples of blood were attained from the KDA hospital Kohat, KPK, Pakistan. The EDTA tubes were used to analyze the samples at 4° temperature.

#### 2.4.2. Parasite Staining and Visualization

The gold standard for diagnosing malaria is still simple and direct microscopic observation of the blood specimens to observe the parasites of malaria. For microscopic diagnosis, thick and thin blood films are stained on a glass slide to visualize the malaria parasite. Leishman stain is being used to stain the parasites (0.15%). The percentage, morphological stage, and species of the parasites can be determined by light microscopy of the blood film. In addition to diagnosing malaria, a blood smear can provide useful prognostic information, such as parasite count, the number of circulating pigments, etc.

#### 2.4.3. In Vitro Cultivation of Parasites

Fresh group O^+ve^ human erythrocytes were dissolved at 4% hematocrit in complete RPMI 1640 medium, containing 125 mM HEPES, 1.11 mM glucose, 0.2% sodium bicarbonate, 0.5% Albumax I, 45 μg/L hypoxanthine and 50 μg/L gentamicin under a gas mixture 5% O_2_, 5% CO_2_, 90% N_2_ and incubated at 37 °C for maintaining the culture. The prepared culture medium was replaced with a completely fresh medium every day to propagate the culture. The parasitemia was monitored using a microscopic examination of Giemsa-stained blood smears. The 5% sorbitol treatment resulted in synchronized ring stage parasites [27].

#### 2.4.4. Drug Dilutions

Milli-Q water was applied to prepare the stock solutions of chloroquine (CQ), FeCl_3_·6H_2_O, plant extract, and FeO NPs. To obtain the required drug concentrations, all stocks were diluted with a culture medium. The drug solution and FeO NPs were then located on 96-well tissue culture grade flat bottom plates (Corning, NY, USA).

#### 2.4.5. Antiplasmodial Activity 

The antiplasmodial activity of synthesized FeO NPs was investigated against the Plasmodium parasite. Sorbitol-synchronized ring stage parasites were incubated in the presence of FeO NPs at a concentration of 25 g/mL under standard culture conditions. For positive controls, CQ was used. After that, a multi-channel pipette was used to integrate the FeO NPs to each well twice for 48 h at 37 °C in the dark. The Victor Fluorescence Multi-well plate reader (Perkin Elmer, Rodgau, Germany) was to measure the fluorescence along with the excitation and emission wavelengths. The counts in each well were subtracted from the fluorescence counts for CQ. Fluorescence counts for CQ were subtracted from counts in each well. The fluorescence counts were plotted against the drug concentration, and IC_50_ (the 50% inhibitory concentration) was determined by analysis of dose–response [28].

### 2.5. Cytotoxic Activity of FeO NPs 

#### Cytotoxic Evaluation

The MTT assay (3-(4,5-dimethylthiazol-2-yl)-2,5-diphenyl tetrazolium bromide) was carried out to check the cytotoxicity of the synthesized FeO NPs, which were slightly modified from a previous study [29]. Briefly, 25,000 PBMCs were incubated for 24 h in a 96 well plate containing RPMI 1640 media supplemented with final concentration FeO NPs at the range of 5–25 µg/mL. The control group consisted of PBMCs that had not been exposed to FeO NPs. The PBMCs were centrifuged and washed with sterile Phosphate buffered saline (PBS) after 24 h of incubation. The PBMCs were then incubated for four hours with an MTT solution. After adding dimethyl sulfoxide (DMSO), the absorbance at 570 nm was recorded by utilizing an ELISA reader (Thermo Fisher Scientific Inc., Waltham, MA, USA). The statistical analysis was done as a percentage (%) viability of PBMCs exposed to FeO NPs compared to a control group that did not receive any treatment.

## 3. Results and Discussion

Several researchers emphasized and demonstrated the potential for shade-dried plants to be used as a stabilizing and reducing agent for the formation of nanoparticles. The accumulation of 1 mL (0.01 M Fe solution) was added to the 9 mL solution of *N. exaltata* plant extract. After some time, the greenish yellow color of the solution changed to reddish brown, indicating the synthesis of FeO NP. Furthermore, the UV–Vis spectroscopic analysis was used to validate the synthesis of FeO NPs, and the spectrum revealed a prominent peak after 1 hour of reaction. This spontaneous reduction reaction is facilitated by the reaction of polyphenolic compounds in the plant extract with an iron-containing solution [30]. 

### 3.1. UV-Visible Analysis of FeO NPs

**Figure 1:**
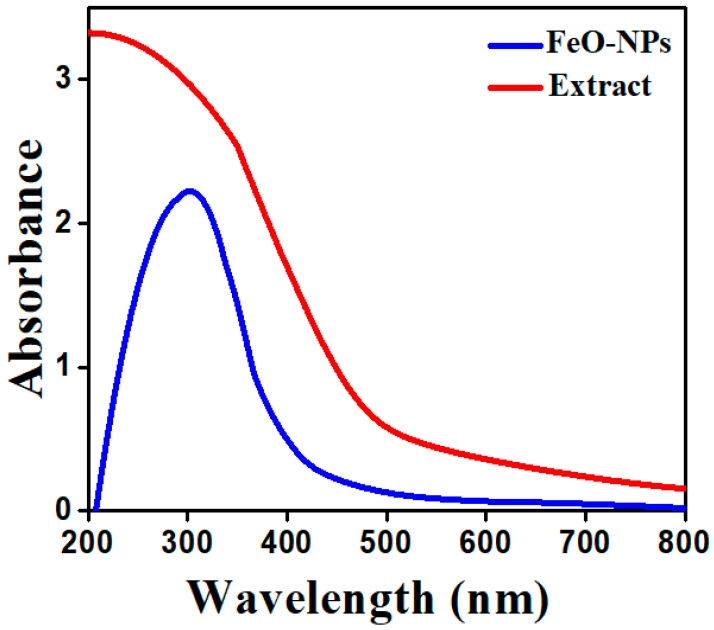
UV-Vis spectra; Red curve for *N. exaltata* plant extract and Blue curve is for synthesis of FeO NPs, maximum yield was obtained at 1: 9 (*v*/*v*) plant extract with 0.01 M FeCl_3_·6H_2_O solution after 2 h incubation at 60 °C temperature.

The peak absorption in the visible region of the spectrum ranges between 300 and 800 nm was investigated using UV-Vis analysis. It practices near-infrared and visible light to work. The visible range absorption peak was significantly influenced by the pigment of the solution in the reaction mixture. The transition of electrons takes place in molecules in a specific region of the electromagnetic spectrum. Figure 1 depicts the spectroscopic UV-Vis analysis of FeO NPs in a mixture containing an aqueous extract plant of *N. exaltata* and precursor. The absorption peaks at 294 nm indicate the formation of FeO NPs [31]. This is because valence electrons of nanoparticles absorb radiant energy in their atomic state and undergo higher energy level transition. The Equation (1) below was used to calculate the energy band gap of nanoparticles. The synthesis of FeO NPs is demonstrated by the broad absorption band at 294 nm, as shown in Figure 1.

E = *hc/*λ (1)

where E corresponds to the band gap energy, Planck’s constant (6.626 × 10^−34^ kg m^2^/s) is represented by *h* and *c* denote the light velocity (3 × 10^8^ m/s), and the wavelength (294 nm) of the nanoparticles is represented by λ. The 4.22 eV was determined by calculating the band gap energy for the NPs at 294 nm. Previously, at 233 nm, a similar result was reported [31].

### 3.2. FT-IR Analysis of FeO NPs

**Figure 2:**
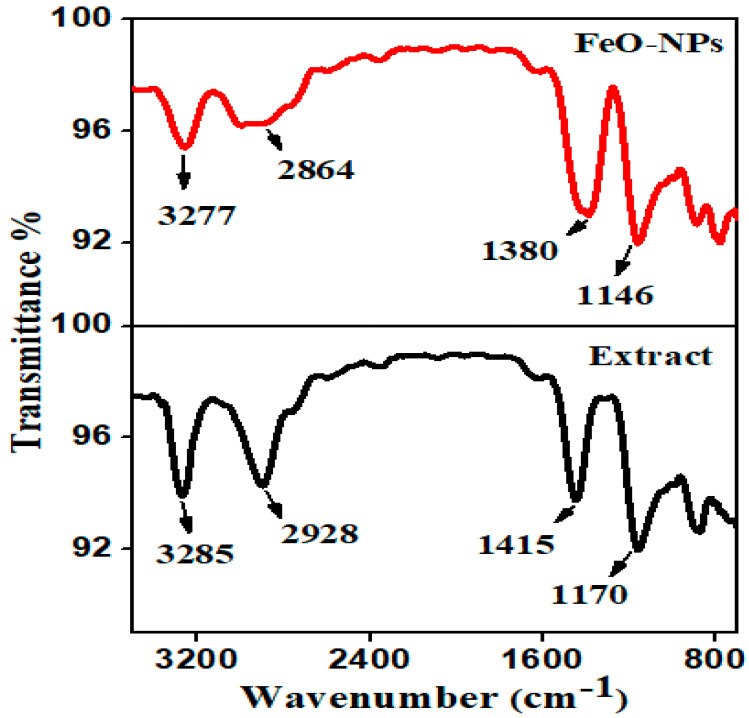
FT-IR spectrum for the identification of functional groups in aqueous plant extract *N. exaltata* and synthesized FeO NPs.

The synthesis of FeO NPs was studied using FT-IR (Bruker, Alpha-II, Osaka, Japan) spectroscopy. The mention Figure 2 illustrates the FT-IR spectra of FeO NPs synthesized and plant extract. An aqueous plant extract contains biologically active functional groups and interaction sites that act as stabilizing, capping, and reducing agents. The occurrence of phytochemical constituents such as phenols, alkanes, carboxylic acids, alcohols, aldehyde, and aromatic compounds was revealed by FT-IR analysis. Among them, the most distinctive band of OH stretching vibration of the phenolic group was obtained at 3285 cm^−1^. The CH stretching vibration is characterized by the apparent band at 2928 cm^−1^. The peak at 1415 cm^−1^ demonstrates the stretching vibration of C-C groups derived from aromatic rings in the plant extract. The band at 1170 cm^−1^ may be referred to as the symmetrical vibration of C–O related to a C–O–SO_3_ functional group [32]. The occurrence of metal-oxygen (Fe–O) bonds is attributed to the small peaks at around 400–700 cm^−1^ [21]. The presence and shifting peaks in the FeO NPs after FeCl_3_ reduction indicate interaction among the functional groups of the plant extract and the iron salt precursor. The reduction and stabilization of FeO NPs are aided by this interaction.

### 3.3. XRD Pattern of FeO NPs

**Figure 3:**
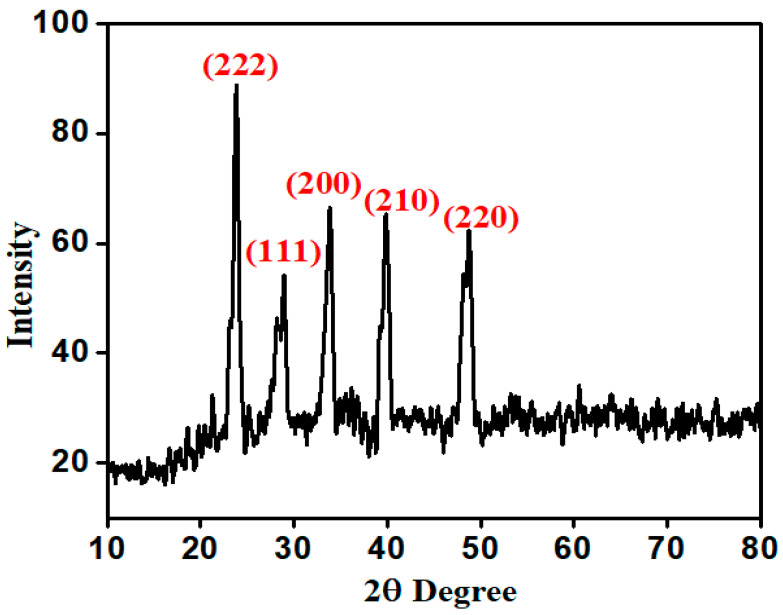
XRD patterns for synthesized FeO NPs from the plant extract, showing a different peak at 2θ degree, indicating the crystallinity of synthesized nanoparticles.

The XRD technique is important for assessing the crystal structure and phase of FeO NPs. The synthesized FeO NPs from *N. exaltata* showed a significant XRD pattern, as mentioned in Figure 3. The five distinct peaks in the XRD pattern are 23.6°, 28.7°, 33.9°, 39°, and 48.6° at 2θ and are marked by their respective miller indices (222), (111), (200), (210), and (220). The high peak intensity of such crystal planes is strikingly similar to the orthorhombic structure. Such findings were compatible with the standard XRD pattern (JCPDS 76-0958). The crystalline nature of the FeO NPs prepared of *N. exaltata* plant extract was evident as a result of XRD analysis. Equation (2) represents the Debye–Scherrer formula, which was applied to calculate the mean diameter of FeO NPs.

D = Kλ/(β cos θ)(2)

where D represents the average particle size of nanoparticles, β identifies the value of the full width at half maximum (FWHM) in the lines of X-ray diffraction, λ signifies X-ray radiation source wavelength of 0.15405 nm, θ is the half angle of diffraction (Bragg angle), and the Scherrer constant with a value from 0.9 to 1 is denoted by K. The high-intensity peak was used to determine the mean diameter of particles, which was found to be 16 nm [33].

### 3.4. SEM Analysis of FeO NPs

**Figure 4:**
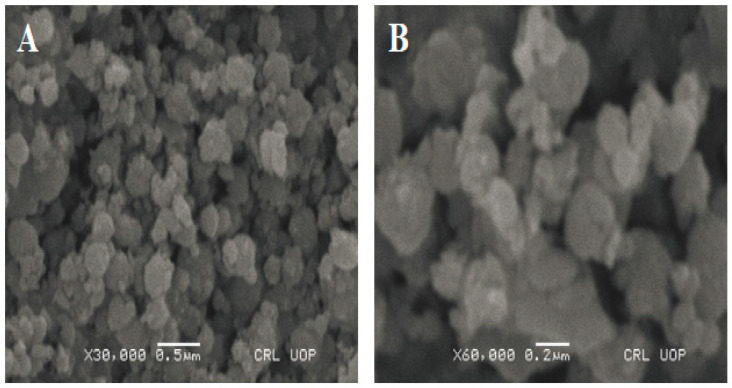
SEM images for FeO NPs. (**A**,**B**) demonstrate isolated spherical and ellipsoidal nanoparticles of different sizes under the magnification of 0.5 and 0.2 μm.

The SEM analysis was carried out to determine the geometry and size of particles. The following figure (Figure 4a,b) shows the roughly spherical and partially dispersed shape of the synthesized FeO NPs. According to the findings, the interaction between the magnetic nanoparticles leads to the aggregate of the FeO NPs to some extent. The average diameter of FeO NPs ranges from 30 to 70 nm [34]. The production of nanoparticles with agglomeration was indicated by the highly conglomerate form of nanoparticles. The aggregation of FeO NPs was observed in SEM images, which could also be due to the evaporation and removal of solvent used during sample preparation. Solvent removal causes electrostatic forces to bring FeO NPs closer together and form aggregation [35,36].

### 3.5. EDX Analysis of FeO NPs

**Figure 5:**
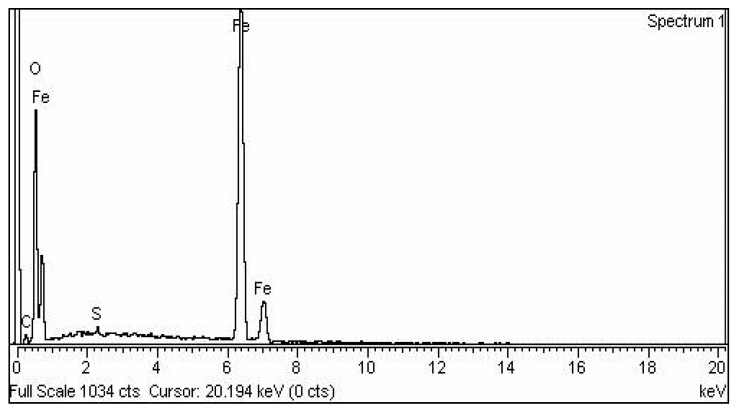
EDX Analysis of FeO NPs to detect the elemental compositions of synthesized nanoparticles at 20 keV accelerating voltages.

The elemental composition of synthesized FeO NPs was ascertained through EDX analysis. The mentioned spectra (Figure 5) showed distinct peak oxygen (O) and iron (Fe), indicating the formation of FeO NPs. A similar detection of Fe and O in phyto-fabricated FeO NPs has also been reported in previous literature [37]. The high-yield Fe element was observed in the L line related to the binding energies of biologically synthesized nanoparticles with peaks around 0.7 and 6.4 keV. The presence of other elements in the EDX spectrum, such as S and C, was most likely due to X-ray excitation from the used SEM grid, precursor salt, and unintentionally inserted from the surface molecule of the plant extract.

### 3.6. Antiplasmodial Evaluation 

**Figure 6:**
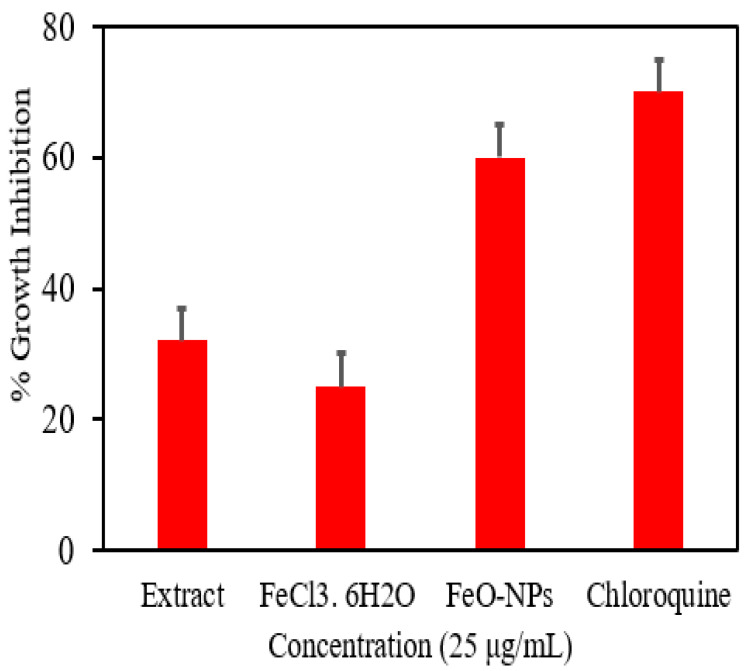
**%** Growth inhibition with plant extract of *N. exaltata*, Precursor (FeCl_3_·6H_2_O), FeO NPs, (standard) Chloroquine for positive control.

The antiplasmodial activity of FeO NPs synthesized from plant extract of *N. exaltata* was observed by the rate of inhibition of malaria parasites. A significant rate of inhibition was observed by assessing the optimum concentration of FeO NPs at 25 µg/mL. The synthesized FeO NPs were examined for inhibition of Plasmodium parasites. The purpose of the current investigation was to evaluate that synthesized FeO NPs were inhibiting the growth of Plasmodium parasites. The attained outcomes confirmed that the synthesized FeO NPs exhibited an excellent efficiency towards the growth of parasites, as mentioned in the IC_50_ values for FeONPs antiplasmodial activity in Table 1. Hence, the data suggest that FeO NPs were found to be more prevalent against Plasmodium parasites, which showed 62 ± 1.3 parasite inhibition at 25 μg/mL. The results confirmed that the FeO NPs exhibit an excellent inhibitory activity when compared to the precursor (FeCl_3_·6H_2_O) and plant extract *N. exaltata*. The control group (chloroquine) showed 70% growth inhibition, while *N. exaltata*-synthesized FeO NPs inhibit 62%, *N. exaltata* plant extract showed 35%, and precursor FeCl_3_·6H_2_O was 23%, respectively (Figure 6). The current findings are comparatively consistent with those of the previously reported literature on the potential antiplasmodial activity of synthesized silver nanoparticles using leaves extracts of *Catharanthus roseus* Linn, with IC_50_ values being 20 ± 0.7% at 25 µg/mL and 75 ± 1.1% at 100 µg/mL [38].

### 3.7. Biocompatibility Assessment

As the production and application of nanoparticles increase, the number of reports implying nanoparticle toxicity in both environmental and medical aspects also increases [39]. Therefore, in this study, we tested the toxicity of the synthesized nanoparticles on human PBMCs in vitro experiments.

#### Cytotoxic Activity

**Figure 7:**
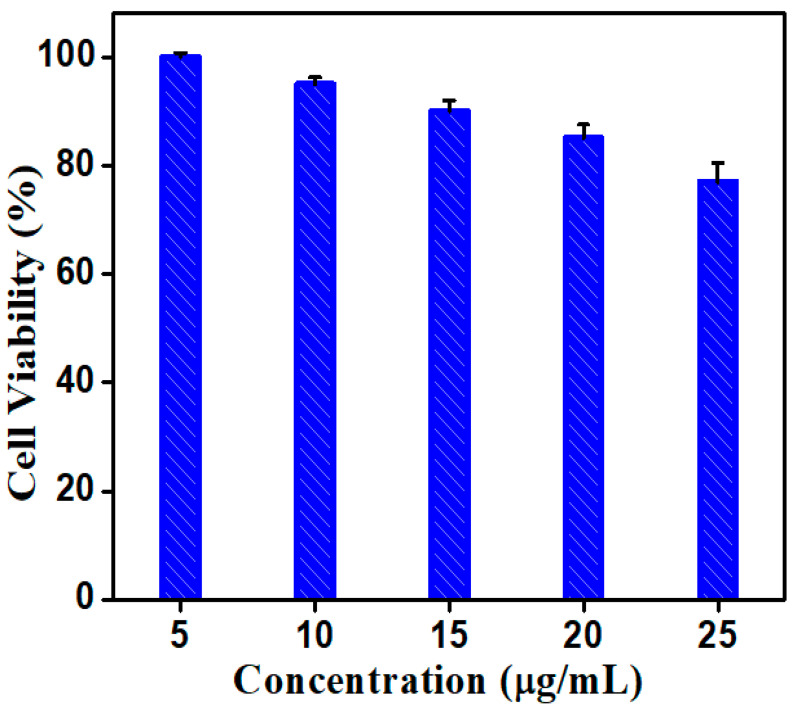
% Cell viability of FeO NPs on PBMCs (round-nucleated cells) at different concentration (μg/mL).

PBMCs are round-nucleated cells obtained from blood or buffy coats that are easily accessible. The two major drawbacks of chemotherapeutic drugs are drug resistance and systemic toxicity. Human PBMCs have been widely used for cytotoxicity testing due to their susceptibility to new drugs and chemicals [40]. The cytotoxic activity of FeO NPs on PBMCs was thus determined using the MTT assay. When PBMCs were treated with different concentrations of FeO NPs (5, 10, 15, 20, and 25 μg/mL), we discovered that their cell viability did not change significantly. On the other hand, the cell viability in PBMCs treated with FeO NPs was observed to reduce as the concentration was increased. Figure 7 illustrates the cell viability percentage against different concentrations. The accomplishment of FeO NPs on PBMCs showed 78% viability at 25 μg/mL, respectively. The action of FeO NPs is considered to be non-toxic when cell viability is greater than 70%, as per ISO 10993-5: 2009 [41]. As a result, the FeO NPs that were synthesized can be considered non-toxic. Other studies did not exhibit cytotoxicity in normal cells treated with green synthesized nanoparticles, which supports our findings [42]. The absence of toxicity in FeO NPs provides more stability for subsequent pharmaceutical and other medical applications.

### 3.8. Proposed Mechanism for FeO NPs 

**Scheme 1:**
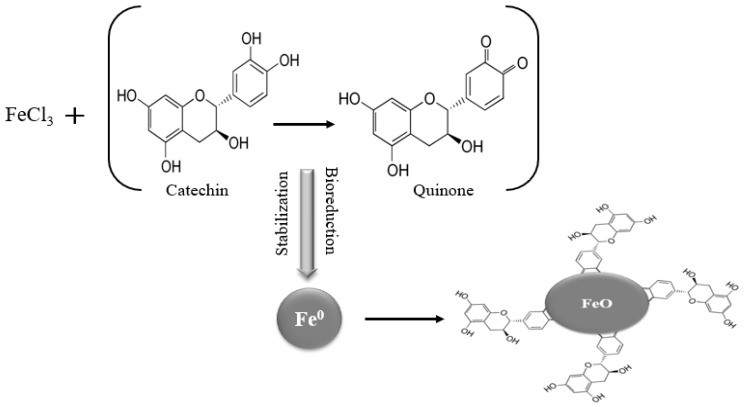
Proposed mechanism for synthesis of FeO NPs.

According to the literature [25], *N. exaltata* plant extract contains a variety of phytoconstituents, including saponins, steroids, alkaloids, phenols, and tannins (catechin) that act as reducing and stabilizing agents. Due to the complicated chemical components of plant extracts, it is difficult to determine the exact mechanism of FeO NPs using plant extract. However, a possible mechanism is predicted in light of the chemical composition of *N. exaltata* mentioned above. The catechin groups belong to tannin molecules present, have a high density of phenolic-OH groups, and are capable of taking part in redox reactions to form quinones. The liberation of electrons makes it possible for the reduction of the iron salt, allowing the formation of FeO NPs, because of the chelating action of adjacent hydroxyl groups in tannins. The formation of the H^+^ radical is attributed to the electron losing property of catechin, which results in the reduction of Fe metal ions into FeO NPs. Based on such results, a possible reaction mechanism involved in the green synthesis of FeO NPs using plant extract as reducing and stabilizing agents is summarized in Scheme 1. Hence, the stabilized FeO NPs are synthesized by a given reduction reaction. Based on such results, a possible reaction mechanism involved in the green synthesis of FeO NPs using plant extract as reducing and stabilizing agents is summarized in Scheme 1. Therefore, the synthesis of FeO NPs was achieved by various biomolecules present in aqueous plant extract that act as reducing and stabilizing agents [43].

## 4. Conclusions

In the current research work, a green synthesis approach was used to synthesize FeO NPs that were cost-effective and environmentally friendly. The aqueous extract of *N. exaltata* encompasses sufficient biomolecules, which serve as both an antiagglomeration and reducing agent. Colloidal FeO NPs were examined by means of UV-Vis, XRD, FT-IR, SEM, and EDX analysis. The average diameter of FeO NPs was 16 nm with their spherical geometry making them useful as therapeutic agents. The nanoparticles showed an effective antiplasmodial agent against a range of Plasmodium parasites. The synthesis of FeO NPs exhibits significant inhibition against the parasites as compared to extract and precursor. However, the biocompatibility (cell viability %) of FeO NPs was shown to have higher cell viability at 25 μg/mL, which can be used as new and safer antiplasmodial drugs.

## Figures and Tables

**Table 1:** In vitro antiplasmodial activity of plant extract/FeCl_3_·6H_2_O and the synthesized FeO NPs with parasite inhibitory concentration (%).

S. No	Test Samples	Test Sample Concentration (μg/mL)	Parasite Inhibitory Concentration (%)
1	FeCl_3_·6H_2_O	25	23 ± 0.3
2	Plant extract *N. exaltata*	25	35 ± 0.6
3	FeO NPs	25	62 ± 1.3
4	Control	25	70 ± 0.32

## Data Availability

All the available data are incorporated in the MS.
